# Engaging Public Health Critical Race Praxis in Local Social Determinants of Health Research: The Youth Health Equity and Action Research Training Program in Portland, OR—yHEART^PDX^

**DOI:** 10.3390/ijerph19138187

**Published:** 2022-07-04

**Authors:** Ryan J. Petteway, Lourdes A. González

**Affiliations:** OHSU-PSU School of Public Health, Portland State University, Portland, OR 97201, USA; lougonzalez90@gmail.com

**Keywords:** social determinants of health, social epidemiology, youth participatory research, antiracism, public health training, public health practice

## Abstract

The social determinants of health (SDH) have long been considered a core mechanism through which racial health inequities are (re)produced and incubated in the U.S. Moreover, scholars have expressly—and appropriately—named structural racism as a precursor to inequities associated with SDH. However, while research on racial health inequities—SDH-related or otherwise—continues to grow, communities of color remain grossly underrepresented as public health researchers and practitioners. Additionally, although SDH are experienced in a very local sense, much research and practice fails to more deeply and thoroughly engage and center local community knowledges. Thus, much work around SDH and racial health inequities presents, ironically, as structurally racist itself—being done/led mostly by White scholars and in ways that do not “center the margins”. Moreover, in the context of public health practice, youth perspective is seldom centered within local health department (LHD) community SDH assessment efforts. With these challenges in mind, this paper introduces and discusses the development of the youth health equity and action research training (yHEART) program as a model for public health researchers/practitioners to engage public health critical race praxis (PHCRP) to better understand and respond to local SDH in communities of color. Specifically, we highlight the significance of PHCRP principles of “voice” and “social construction of knowledge” in advancing antiracism in research and LHD practice related to local SDH. First, we articulate core conceptual and theoretical groundings that informed the yHEART program’s development and animate its ongoing training and research activities. Second, we outline the program’s core training components and overall process, and provide some brief illustrative examples of work completed during the program’s first iteration—yHEART PDX, Vol.I: Youth Participatory Research on Local Social Determinants of Health. We then close with a discussion that reflects on program strengths, challenges, and implications for SDH and racial health equity research/practice in light of growing calls for an antiracist public health.

## 1. Introduction

The social determinants of health (SDH) have long been considered a core mechanism through which racial health inequities are (re)produced and incubated in the U.S. Moreover, scholars have expressly—and appropriately—named structural racism as a precursor to inequities associated with SDH [1,2,3,4,5,6,7,8,9]. However, while research on racial health inequities continues to grow, people of color still make up less than 25% of tenure-track school of public health faculty [10]. Indeed, just 5.7%, 5.7%, and 0.3% of faculty are Black, Latinx, and Indigenous, respectively. Moreover, communities of color are also grossly underrepresented in grant review and journal editorial board positions [11,12,13], meaning that all stages of the racial health (in)equity research and knowledge production enterprise—SDH-related or otherwise—are disproportionately White. This creates and incubates a context in which people of color are represented/present in racial health inequities and SDH research mostly—and most often—when they are the “objects” of the scientific gaze of researchers, funders, and peer-reviewers whose social locations are markedly different from their own. Thus, SDH and racial health equity knowledge production, as structural racism would have it, is dominated and curated mostly by White scholars. In short, discourse about the critical import of SDH in shaping racial health inequities has failed to account for the structural racism embedded within the SDH research and knowledge production process itself.

A core question that should animate public health efforts to address SDH as germane to racial health inequities is this: what is public health doing to address its own structurally racist self? As has been articulated elsewhere, racism is not just “out there” to be studied—it is also very much “in here” [14,15], where it fundamentally shapes what is deemed worthy of studying, who gets to study it, who tangibly benefits from its study, and how what is studied/learned is translated into practice. Recent scholarship has centered these and related concerns within various domains of public health research and practice [16,17,18,19], yet more work is needed to move us forward and deepen interrogations towards interventions aimed at structural change. For example, how, and why, is it that our multibillion-dollar enterprise has failed to interrogate itself in this regard? How might we manifest a future public health SDH and racial health equity research and practice that is antiracist in principle, practice, and process? How might we work towards an SDH and racial health equity research wherein the *direct* economic and social beneficiaries are those traditionally restricted to being “objects” and data points?

Here, we suggest that part of the response to these questions must include concerted efforts within both public health research and practice to more thoroughly engage antiracist and decolonizing praxes—praxes that “center the margins” [20]. Specifically, we suggest that public health research and practice must do more to create opportunities for the communities experiencing the embodied health consequences of racial and social inequities to be more deeply involved and centered within SDH research/practice processes. Moreover, they must create opportunities for current SDH “n’s” to become future SDH researchers/practitioners. 

In this spirit, this paper introduces and summarizes a program developed with these considerations, tensions, and possibilities in mind: the Youth Health Equity and Action Training program in Portland, OR—yHEART^PDX^. In the following sections, we first articulate core conceptual and theoretical groundings that informed the program’s development and animate its ongoing training and research activities. Second, we outline the program’s core training components and overall process, and provide some brief illustrative examples of work completed during the program’s first iteration—yHEART PDX, Vol.I: Youth Participatory Research on Local Social Determinants of Health. We then close with a discussion that reflects on program strengths, challenges, and implications for SDH and racial health equity research/practice in light of growing calls for an antiracist public health.

## 2. yHEART^PDX^: Conceptual Roots and Theoretical Groundings

yHEART^PDX^ draws from social epidemiology, critical theory, critical race theory, Black feminist theory, decolonial theory, and principles/practices of community-based participatory research (CBPR) and data justice to provide public health training and participatory research opportunities for youth of color. The program is based on an initial yHEART project that was developed based on the *People’s Social Epi* (PSE) framework introduced by Petteway and colleagues [21]. Generally, PSE is a multi-tiered framework for guiding social epidemiology in becoming more inclusive, equitable, and actionable for 21st century practice, integrating social epidemiology theory, principles/practices of CBPR [22,23,24,25,26], and conceptual and technological affordances of information and communication technologies, or ICTs (e.g., smartphones, web-based mapping), anchored in the field of ICT for Development, or ICTD. Core conceptual and theoretical groundings within social epidemiology that informed the development of yHEART^PDX^ included ecosocial theory [27], embodiment [28,29], fundamental causes [30,31], and social production and political economy [32,33,34], as well as scholarship around structural racism [1,2,8,35], environmental justice [3,36,37,38,39,40], intersectionality [41], and aspects of allostatic load, epigenetics, and the lifecourse [42,43,44,45]. As detailed in Petteway and colleagues [21], core concepts related to the use of ICTs that informed the development of PSE and yHEART include liberation technology [46], deliberation technology [47], and small data [48].

The PSE was developed with a particular focus on place-health research and applications within local health department (LHD) community assessment practice, which, as articulated by Petteway and colleagues [21], is “particularly well-suited… to leverage the practical and procedural translational advantages of much place-based research (e.g., space-bound, locality- and/or jurisdiction-specific), while simultaneously capitalizing on the scientific and political translational advantages of harnessing place-based knowledge, insight, and expertise of the people whose lives unfold within the ‘place’ being studied” (p. 6). The initial yHEART project was accordingly informed not only by conceptual place-health work, e.g., notions of “opportunity structures” [49], “relational place” [50], and “spatial polygamy” [51], but also by critical concepts related to agency and knowledge production, including critical consciousness [52], situated knowledges [53], decolonizing methods [54,55], and power and resistance from the margins [56,57]. 

It is this notion of harnessing knowledges and/as power from the margins that animated yHEART development and engagement with public health critical race praxis, or PHCRP [58]. PHCRP extends core tenets of critical race theory (CRT) to public health contexts. Core amongst these for yHEART is “centering the margins” [20], with focus areas of knowledge production and action also salient in its development [58]. As articulated by hooks [57], the margin—while certainly acting as a site of oppression and exclusion in the context of SDH and racial inequities—is also a site of resistance and “radical openness and possibility” (p. 153). Those at the margin have a deep, embodied, and unique knowledge of SDH-related conditions and power structures in their communities, and thus a unique power to name, frame, and act upon them. However, standard public health research and LHD community assessment practices fail to engage those at the margin as knowers and social change agents capable of researching their own lives and communities—habitually foreclosing meaningful engagements with PHCRP principles like “voice” and “social production of knowledge” [58].

yHEART was developed with these two PHCRP principles, “voice” and “social construction of knowledge”, at the forefront. As described by Ford & Airhihenbuwa [58], voice is the, “privileging of marginalized persons’ contributions to discourses” (p. 1396), in such a way that it can help, “illuminate disciplinary blind spots that are otherwise imperceptible from within a discipline’s mainstream”. For yHEART, it was important to arrive at program design that centered youth voice, valued their lived and embodied knowledges, and honored their agency—such that any research related activities engaged them as co-researchers and experts on their own lives/communities, not as “objects” and samples. This of course is directly connected to the PHCRP principle of “social construction of knowledge”—the reality that research is, “inherently subjective and tied to the social context in which it is conducted” [58] (p. 1395). This principle not only highlights the importance of inclusive research practices that view community knowledges as valuable and legitimate, but also necessitates the interrogation of dominant/traditional research and practice processes related to SDH and racial health inequities led by supposedly “objective” outsiders. yHEART was developed to unapologetically center the lived and embodied knowledges of youth of color, and to explore ways in which their knowledges might complement, contextualize, and/or counter those produced by their LHDs. 

LHDs play a critical role in collecting SDH data and assessing the current state of place-based health inequities in the communities they serve. However, many LHDs are confronted with fiscal, political, and jurisdictional limitations that compromise their ability to adequately assess and equitably respond to community SDH concerns. Moreover, youth perspective is seldom included in LHD community assessment efforts. In this regard, LHDs can often function to silence or erase youth knowledges and misrepresent their experiences/lived realities—unnecessarily, and harmfully, curtailing prospects for deeper engagements with PHCRP for meaningful local SDH action. yHEART was thus developed in the belief that LHDs can and must do better to center the margins, and that the integration of ICTs with social epidemiology and CBPR could offer a way to elevate community knowledges, democratize local assessment processes, and mature existing LHD strengths to advance public health practice and policy in relation to SDH [21]. Research activities under yHEART^PDX^, as with initial yHEART project, were accordingly planned with ICT usage in mind.

## 3. yHEART^PDX^ Vol.I: Youth Participatory Research on Local Social Determinants of Health

### 3.1. yHEART^PDX^ Overview

yHEART^PDX^ trains youth of color and low-income youth residing in N/NE Portland as public health researchers, integrating social epidemiology and youth participatory action research (YPAR). Using ICTs to enhance research processes, yHEART^PDX^ has three core goals:

1. Create opportunities for youth to influence local public health practice and policy
2. Assess community SDH through democratized community health assessment processes
3. Provide training opportunities for youth to gain public health skills for future educational opportunities

The first yHEART^PDX^ project—yHEART^PDX^ Vol.1: Youth Participatory Research on Local Social Determinants of Health—examined youth researchers’ place-based experiences of local SDH. In collaboration with Self Enhancement Inc. (SEI) in NE Portland, and community artist and art professor Sharita Towne, the activities under this project had three interrelated research and training objectives:

1. Pilot a curriculum for training local youth on health equity, SDH, and participatory research
2. Guide youth through a participatory research project to identify and map local SDH concerns
3. Use youth research data to generate a series of research and creative arts products that can be used to inform and guide LHD and city planning practices/strategies related to SDH

The purpose of this project was to understand experiences and perceptions of local SDH among youth of color in N/NE Portland. To that end, youth researchers used four participatory methods (described below) to identify, characterize, and map their place-based experiences/perceptions of local SDH. They then generated creative arts research products (based on their data) to use in a series of planned community exhibits and research dissemination events. The goal of this project was to identify specific places where these SDH are located, and work with youth to understand how they affect health opportunities in N/NE Portland and how to intervene. In other words, we wanted to collaboratively map out important youth SDH locations and experiences that could inform youth-centered LHD practice. Additionally, we wanted to generate local SDH data, maps, and arts products that could be used to improve community health opportunities from a youth perspective.

### 3.2. yHEART^PDX^ Training + Research Process

Youth attending SEI’s after school programming were recruited as youth researchers for this project. These yHEART^PDX^ youth attended 7 training sessions covering core principles and concepts related to SDH, health equity, research ethics, participatory research, and “place” and health (Table 1). Youth researchers were then trained in four participatory methods (Table 2). Youth were not able to participate as researchers until they completed these training sessions. Integrating ICTs and YPAR, the youth used these participatory methods, including photovoice (via smartphone) and web-based participatory GIS, to identify and map their place-based experiences/perceptions of local SDH. Then, using X-ray Mapping (described below), youth created symbolic “body maps” reflecting how each place-based SDH they identified affects their bodies. A total of 14 youth completed the trainings and participated in the SDH research project.

Each yHEART^PDX^ training session generally lasted between 90 and 120 min. Youth were provided with a packet of training materials for each session, which included handouts of any presentation slides, any activity worksheets, and a list of the session’s key terms and concepts. Training sessions were structured around use of didactic methods (e.g., PowerPoint) to introduce concepts, with use of interactive activities, full group discussion, and small-group discussions with each group reporting main discussion points back to the full group. Interactive activities included having teams of youth outline a public health response to an SDH issue on a chalk/whiteboard. For example, in discussing SDH as related to the built and natural environment in the *SDH + Social Epi 101 Module*, youth were prompted to identify an important exposure (e.g., air pollution), a related health outcome (e.g., asthma), an important environmental SDH that shapes exposures/risks (e.g., transportation and tree canopy), and how practitioners/researchers in each of public health’s core training and practice areas from the *Public Health 101 Module* (e.g., Epidemiology, Health Education/Promotion, Health Systems and Policy) might intervene. Follow-up discussion explored how aspects of public health law and public health ethics might be relevant. For example, how might administrative law be used to intervene (e.g., issue new regulations)? How might epidemiology data be used to intervene (e.g., advocate for new legislation and build community awareness)? 

In another activity for the *Place*, *Placemaking*, *and Health 101* Module, youth were provided with a list of online public health and SDH databases, including the County Health Rankings [59], CDC 500 Cities Project [60], Big Cities Health Inventory Data Platform, City Health Dashboard, U.S. Life Expectancy Estimation Project, The Opportunity Atlas, and PolicyMap. Individually, they were asked to find the estimated life expectancy for their state, county, ZIP code, and census tract. Then, in small SDH-themed groups (e.g., housing, education), they were asked to identify three SDH indicators for their county and census tract that they believed influenced the life expectancies they found. As a third example activity, for both the *SDH + Social Epi 101 Module* and the *Place*, *Placemaking*, *and Health Module*, youth identified music that they believed spoke to SDH that were relevant to their daily lives. These “public health mixtape sessions” helped to ground youth in their lived experiences and embodied knowledges of SDH, and served to bridge their knowledge to the content covered via the training modules.

While modules were initially developed to be covered in one session, we more often than not divided each module over two or sometimes three sessions. This was mostly in response to two key factors: (1) too much material included in each Module and more discussion than initially anticipated, and (2) changes in SEI’s after school programming schedule that reduced the amount of time available for each session. All training sessions were held either on-site at SEI during after school programming hours, or at a nearby high school (during parts of the summer). Youth received a stipend for each training session they completed.

Once youth completed these training sessions, they were able to formally join the SDH research project, i.e., we collected youth assent and parental consent forms. We made sure to separate the phases/components of the program to maximize the number of potential youth who could at least participate in the training modules, with the understanding that not everyone would want or have time to join the more formal research project. We thus structured our research protocol for our university ethics review board to clearly delineate what constituted youth being engaged as learners, and then becoming involved as active co-researchers—the latter constituting them becoming “subjects” in the view of the ethics board. 

For the yHEART^PDX^ research project component, youth were trained in and used four participatory methods to document their daily place-based experiences and perceptions of local SDH (Table 2). First, they used Photovoice to visually document important place-based SDH experiences/exposures. They attended 7 total Photovoice sessions to complete the method, with each youth selecting 5 final photos to map and develop written narratives for. Youth completed their own participatory coding and theming analysis of their photovoice data. Second, using their final 5 photovoice photo-locations as the starting point, youth completed participatory Activity Space Mapping via a web-based mapping platform. Here, they used markers to indicate the spatial locations of their place-based SDH photos and to identify other important locations not captured via Photovoice. They then completed an Activity Space Worksheet for each of their final 5 photovoice photos. Third, again using their final 5 photovoice photos as the basis, they completed X-ray Mapping worksheets to describe how they perceived each place-based SDH experience/exposure affected their bodies. Lastly, for Participatory GIS, youth integrated all of their SDH data via a web-based mapping platform. Youth received a stipend for each research session they completed.

**Table 2:** Summary of participatory research methods used by yHEART^PDX^ youth researchers.

yHEART^PDX^ Participatory Research Method	Description
**Photovoice**	A participatory action research method designed to facilitate the empowerment of youth and adults through photography [61,62]. Participants used photography to visually document their daily/weekly experiences (and perceptions thereof) of place-based SDH. The visual representations they generated provided the focus for group discussions and documentation of stories and themes examining/uncovering their lived local SDH experiences in NE Portland. Participants used their smartphones for this method. Activities for this method included 1 training session covering method conceptual roots and ethical concerns, 3 photo review sessions, and 3 participatory photo analysis sessions. For these analysis sessions, youth were trained to complete their own qualitative coding and theming analysis using a process similar to that detailed in Petteway and colleagues [63].
**Activity Space** **Mapping**	A process by which a participants’ daily activity locations and patterns are mapped out [64,65,66]. In this project, we used a participatory approach. Participants documented their daily activity spaces in a two-fold process. First, they used web-based maps to indicate locations of photovoice photos and other important daily places not captured via Photovoice. Second, they completed Activity Space Mapping worksheets for each photovoice photo location. These worksheets included a series of short descriptive questions and a “Rate Your Place” activity for youth to assign a star-rating to each place (see Figure 2 below). Activities for this method included 1 training session and 1 mapping session.
**X-ray Mapping**	A cognitive mapping method to understand how participants perceive their daily experiences with place-based SDH and how those experiences affect their bodies/health [29,67]. Essentially, a participatory method to capture subjective notions of embodiment. Participants used “X-ray Map” worksheets containing a basic body outline with ventral and dorsal representation on the front side of the paper and were instructed to locate their perceived place-embodiment effects for each SDH photovoice location (see Figure 3 below). They used color-coded stickers, with green representing a perceived positive body effect, red representing a negative effect, and yellow representing both a positive and negative effect. Participants used the back of their X-ray Map worksheets (and often the front) to write a brief description/narrative explaining their SDH place-embodiment representations. Activities for this method included 1 training session covering notions of embodiment, allostatic load, and weathering, as well as method details, and 1 mapping session.
**Participatory GIS**	A method by which participants actively define and spatially locate places and share power in creating mapped realities [68,69]. Here, participants integrated Photovoice, Activity Space Mapping, and X-ray Mapping findings for the creation of web-based maps with photos and narratives embedded into each SDH location they identified. Activities for this method included 2 mapping sessions.

### 3.3. Illustrative Examples of yHEART^PDX^ Vol.1 Research

#### 3.3.1. Photovoice

**Figure 1:**
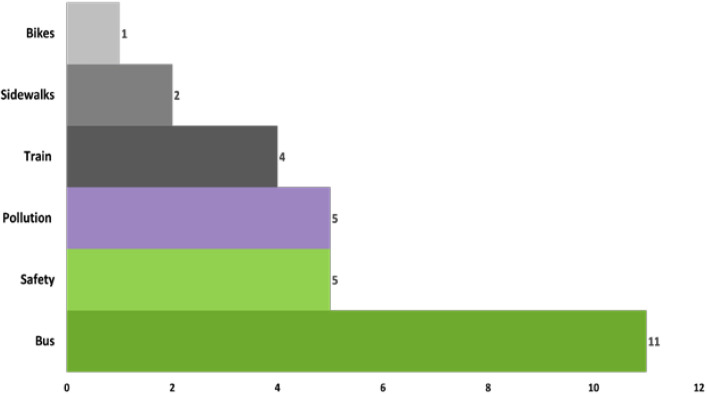
Summary of photovoice photos related to transportation.

For photovoice, each youth researcher selected up to 5 photos to include in their final qualitative and mapping analysis—for a total of 63 photos. Across these photos, 25 (40%) reflected some aspect of transportation opportunity or risk—11 (44%) related to bus infrastructure, 5 (25%) related to safety concerns, and 5 (25%) related to air pollution/environmental health (Figure 1). 

#### 3.3.2. Activity Space Mapping

**Figure 2:**
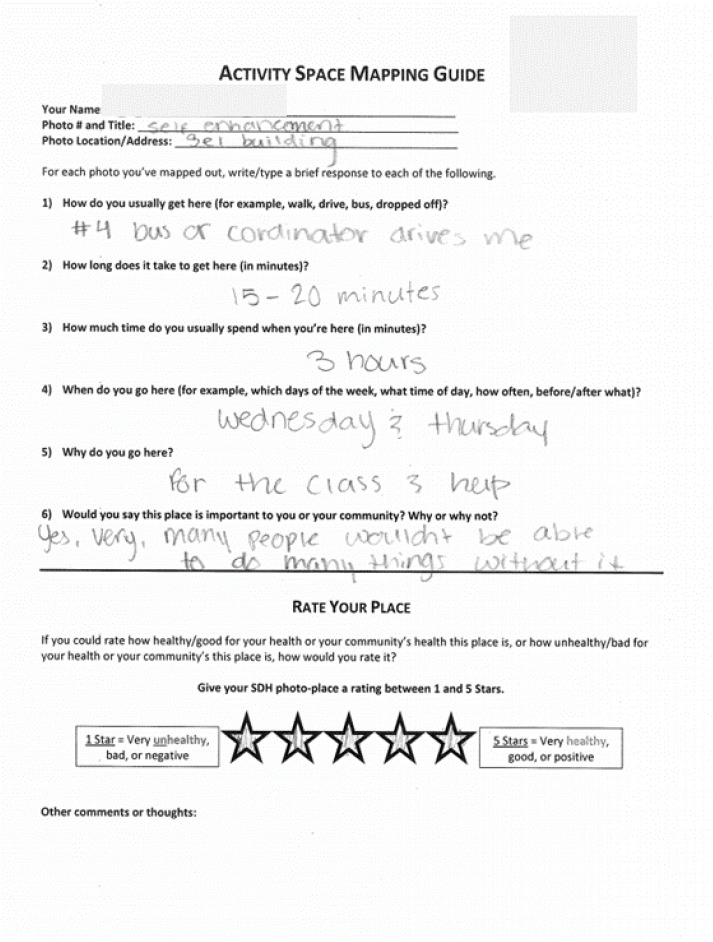
Example of a completed Activity Space Mapping Worksheet.

Youth completed a total of 46 Activity Space Mapping Worksheets. An example is shown in Figure 2 below. 

#### 3.3.3. X-ray Mapping

**Figure 3:**
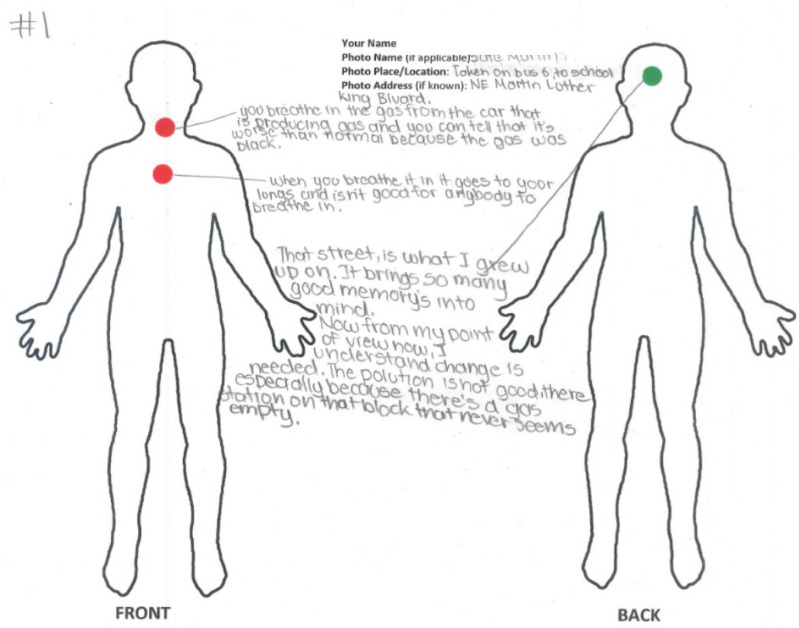
X-ray Mapping example produced by a youth researcher describing where and how place-based SDH affect their body.

Youth completed a total of 50 X-ray maps. An example is shown in Figure 3 below. 

#### 3.3.4. Participatory GIS

**Figure 4:**
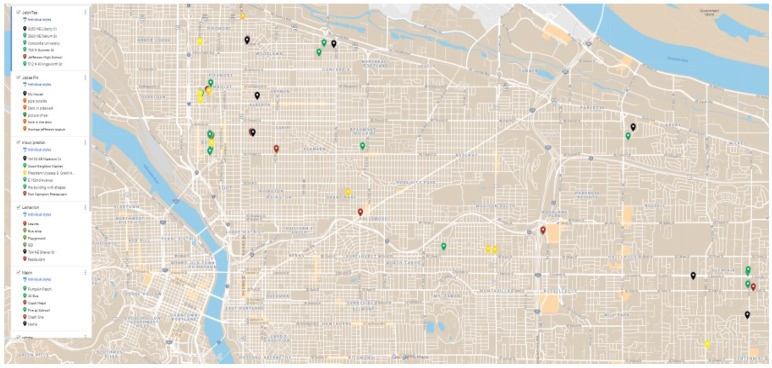
A summary Participatory GIS map showing the locations of some of the daily place-based SDH experiences youth identified. Green = “positive/healthy/good”, Red = “negative/unhealthy/bad”, and Yellow = both “positive” and “bad”.

Each youth researcher created their own web-based map to upload and spatially represent/present their SDH data from Photovoice, Activity Space Mapping, and X-ray Mapping. Data from these individual maps were aggregated into a summary map. The map in Figure 4 below captures some of these aggregated SDH data.

### 3.4. Creative Arts Products and Dissemination

**Figure 5:**
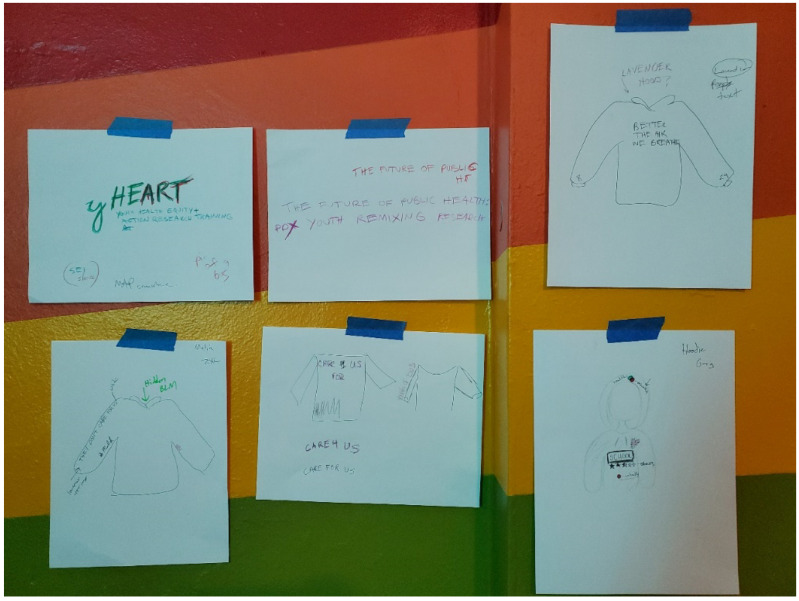
Draft illustrations of some of the youth researchers’ custom-designed hoodies.

Youth researchers worked with local artist and art professor Sharita Towne to transform their SDH research data/findings into creative arts products. They attended three intermittent arts training and discussion sessions to get oriented to art as a social practice and mode of creative resistance, rearticulation, and public discourse, and to more generally discuss their ideas for what they might want to develop—as individuals and collectively. They then attended three or four sessions to develop their creative arts products, joining Sharita Towne in her NE Portland arts studio where she guided them through various production activities. This included work to develop a project zine featuring youth photovoice photos, narratives, and maps; custom buttons with various quotes/phrases based on their research findings; and custom-designed hoodies also featuring quotes/phrases that captured core themes from their research (Figure 5). Youth also co-planned a series of public exhibits which were to feature, among other components: (1) a map projected on a wall showing their X-ray Mapping data for place-based embodiment of SDH, (2) an artistic rendition of all of their X-ray Mapping worksheets, (3) a pair of mannequins wearing custom-designed hoodies that attendees could place pins in to indicate how their own neighborhoods affect their bodies, and (4) display of zines and other products created. Unfortunately, the exhibits were never held due to the COVID-19 pandemic, and many of the planned arts products were left uncompleted due to our university’s required freezing of all in-person research and community engagement activities.

Four youth researchers also helped to prepare an abstract for submission to the 2020 *Youth. Tech. Health Live* conference, to be held in San Francisco, CA. The abstract was accepted and the youth helped to develop a presentation to share their work with an international audience of fellow youth researchers and YPAR practitioners. Unfortunately, again due to COVID-19, we were not able to travel to San Francisco to attend in person, and had to present virtually instead. The youths’ research was also used by the LHD’s Racial and Ethnic Approaches to Community Health (REACH) program to inform some of their youth-centered work around transportation safety and equity (for details of data shared with REACH, see [70]). However, the youth were not able to share their work in-person with the REACH program staff due to the pandemic.

## 4. Discussion

The goal of this paper was to introduce a model program for how to more intentionally and thoroughly “center the margins” within local SDH research and practice. The yHEART^PDX^ program, we believe, does well to illustrate how that might be achieved through engaging PHCRP. Additionally, we also believe the program does well to demonstrate the value and potential impacts such intentional engagements can have for efforts to identify and respond to local SDH concerns. Having said that, we suggest perhaps four core reflections/takeaways regarding program strengths from the first iteration of yHEART^PDX^ in regard to engaging PHCRP.

First, a fundamental aim of yHEART^PDX^ is to unapologetically center the voices of youth of color within local public health research/practice efforts. For yHEART^PDX^ Vol.1, we selected methods and analysis processes that literally and figuratively kept their voice at the center of all research activities. For Photovoice, as noted above (Table 2), youth not only chose what to take photos of, i.e., which SDH and which locations, but they also analyzed their own photovoice narratives via participatory coding and theming. In this way, youth retained narrative control throughout the entire process. In traditional photovoice practice, outside researchers strip agency—and ironically, voice—away at the final analysis stage when they analyze participants’ photos themselves—alone—using qualitative analysis software, then “member check” to see if they got it right. Not only did we think that the traditional approach ran counter to the PHCRP principle of “voice”, but that it also contravened the principle of “social construction of knowledge”—in that it surrenders power of knowledge interpretation and meaning-making to outsiders viewing the data/findings from disparate social locations relative to participants. 

Similarly, we chose X-ray Mapping deliberately as a qualitative method to elicit *subjective* notions of place-based SDH embodiment. As discussed by Petteway and colleagues (2019), research related to embodiment has been overwhelmingly quantitative and oriented around collecting surveys and biological samples (e.g., cortisol) from people. These samples are then used tell (reductionist empirical) stories about participants’ bodies/health—without participants having an opportunity to shape the embodiment narrative at all. The fully participatory X-ray Mapping method, on the other hand, honors PHCRP principles of voice and social construction of knowledge by enabling the youth themselves to tell their own stories about their lived SDH embodiment experiences/perceptions—effectively decolonizing narratives about their place-embodiment. 

Second, we were deliberate in developing alternative ways for youth to engage in analysis and discourse of local SDH, namely, through use of creative arts. Arts play a critical role in public health [71,72,73,74,75], yet they are very rarely engaged as a part of standard research/practice, related to SDH or otherwise. By creating and holding space for creative expression of their lived SDH knowledges through planned research-arts sessions, we believe we not only more deeply engaged PHCRP principles of voice and social construction of knowledge, but honored broader CRT notions of storytelling and counterstorytelling [76,77].

Third, we planned from the beginning to identify ways for yHEART^PDX^ Vol.1 youth researchers to share their voice and knowledges in important community, research, and practice spaces. We nurtured relationships with LHD practitioners to connect youths’ work to important ongoing community conversations around local SDH (e.g., transportation equity and safety), and we remain in conversation with LHD REACH leadership to continue connecting yHEART to local practice in future projects. We also guided youth through the process of preparing an abstract to an international youth health conference. For us, pursuing opportunities for the youth to further amplify their voice and knowledges as legitimate represented crucial avenues to further honor PHCRP. 

Lastly, and more generally, a core strength of the yHEART^PDX^ program is its emphasis on centering youth perceptions and experiences as local subject matter experts and researchers. The program aims to countervail existing legacies of oppression and exclusion in public health practice and research by uplifting local and lived knowledge on SDH, decolonizing the research/practice enterprise, and encouraging new models of community practice for LHDs. We believe our intentional use of ICTs for yHEART^PDX^ Vol.1 greatly facilitated this. Such an approach opens the possibility of technologically and socially evolved LHD assessment practices that reflect commonly articulated values of equity and inclusion. Data from processes/programs like yHEART^PDX^ Vol.1 can be used to enhance, nuance, and contextualize other data sources to advance understanding of community SDH as experienced by residents—and not simply as calculated by local and state administrators/epidemiologists. Importantly, this sort of community-led participatory approach is gaining traction in Oregon via the Oregon Health Authority’s survey modernization and strategic data planning initiatives, which further positions the yHEART^PDX^ Vol.1 project as a potentially instructive model for state-wide efforts. Moreover, such processes could present an important opportunity to leverage collaborative LHD assessments as a mechanism to build community power and increase civic engagement around matters of equity. As such, we believe yHEART^PDX^ Vol.1 did well to illustrate how LHDs might engage PHCRP principles of voice and social construction of knowledge to advance the PHCRP area of action. 

Having said that, yHEART^PDX^ Vol.1 also had its share of challenges and limitations, of which two stood out the most. First, youth participation and commitment throughout the lifecycle of the program was challenging due to term-by-term changes in youth academic and after school schedules. yHEART^PDX^ was implemented as part of SEI’s after school programming; thus, whenever school schedules changed, so did after school programming. Moreover, yHEART^PDX^ Vol.1 training sessions had to be held in multiple locations during the first year—moving from classrooms at a local high school in the Summer terms, to the main SEI building for the remainder of the terms. During the initial summer, about 30 students attended the first few trainings. By the end of the summer—as schedules and training locations changed—only about 15 attended the training sessions. Furthermore, new youth joined the program at various training stages. yHEART^PDX^ Vol.1 was designed to work with a stable cohort of youth. By the time the training sessions were completed and the program moved into research activities, there was a stable cohort of 14 youth—of which maybe 3 or 4 joined at the very beginning. Thus, the ebb and flow of group size and the intermittent addition of new youth presented as a challenge to project momentum, group cohesion, and youth/outsider rapport building.

Second, the COVID-19 pandemic significantly disrupted several key aspects of the program. Youth had completed all research methods by the time shutdowns began and our university froze all in-person research activity. However, they did not have a chance to debrief and discuss their complete and integrated data. They also needed a few more Participatory GIS sessions to fully upload and merge the data from the various methods. Additionally, youth were not able to complete the hands-on sorting and ranking process for deliberating photovoice action, nor did they have a chance to frame their photos for display at exhibits. Additionally, of course, there were no photovoice exhibits. Similarly, youth were not able to complete the development of their creative arts research products, meaning that their art was left in draft/a state of partial completion. Additionally, again, of course, we were not able to host project research-art exhibits as initially planned. Youth were also not able to formally present their work in-person to LHD officials. Finally, we had to cancel plans to travel to San Francisco for five youth researchers to present their work at the 2020 *Youth. Tech. Health. Live* conference. As noted above, four youth still presented virtually. While the youth were still very much excited to present their work, overall, the pandemic necessitated what felt like a premature and anticlimactic close to the project. Additionally, we believe the disruption significantly curtailed its potential impacts related to dissemination and possible follow-on action or follow-up projects. 

Reflecting on these two core limitations as we plan the next iteration, we believe it is best to start with a smaller “core” group of youth co-researchers and focus on their sustained engagement as we expand to include more youth. For the next iteration, we are planning to work with five youth to guide through the training modules, taking more of a workshop approach to allow for deeper material engagement and flexibility. The hope is that this set of youth will not only commit to the longer progression of the yHEART project, but serve as a stable set of peer-mentors/peer-trainers for including additional youth going forward. Additionally, given the continued risks and disruptions presented by COVID-19, we plan to work via a hybrid program model—conducting training modules virtually (synchronous) and facilitating research-related sessions in-person. However, because we are planning to work with a core group of just five youth initial for the second iteration, we are exploring the possibility of having all training sessions in-person as well. Additionally, given the LHD connections established via the first iteration, we hope to involve REACH program staff in the training and research aspects as relevant and mutually beneficial, with ongoing conversations already in progress. Lastly, the second project iteration will include three training workshops focused specifically on decolonizing methods and data justice for public health, to be facilitated by a LHD senior epidemiologist and two researchers who lead research justice work for a local community-based organization.

Overall, we believe yHEART^PDX^ Vol.1 does well to model how to engage PHCRP to center the margins within local SDH-focused work, and perhaps, how to begin training up the next generation of SDH researchers and practitioners of color. Current researchers/practitioners who engage YPAR and/or focus on SDH might consider yHEART^PDX^ Vol.1 as a sort of guide to pursue similar efforts. While we were not able to shore up the formal connections to our LHD for the specific youth in this iteration due to the pandemic, we anticipate doing so going forward, and believe that a program like this might be of particular interest and value to other LHDs that have committed to community-centered practices for addressing SDH and structural racism. While youths’ data/knowledge contributions were neither exhaustive nor representative of all local SDH knowledges, the same can be said of standard LHD community assessment and health surveillance practices. Yet, the latter is given unquestioned credence, often without scrutiny or structures to hold LHDs accountable for matters of inclusion and (mis)representation related to local SDH. Movement to include projects like this within standard LHD practice could prove valuable in ensuring PHRCP principles gain and sustain traction within important community assessment and surveillance practices/processes. Moreover, it could lay the groundwork for growing an inclusive and representative future SDH research/practice workforce—an opportunity to intervene early on the structurally racist SDH and racial health inequities enterprise. 

## 5. Conclusions

The yHEART^PDX^ program models a way to more intentionally and thoroughly “center the margins” within local research and practice on SDH within communities of color. Moreover, it offers guidance on how to begin to pursue long-term changes to build a more inclusive, more representative body of future SDH scholars and practitioners. In a more direct and applied sense, yHEART^PDX^ illustrates how everyday ICTs can be repurposed to uplift community voice and center local knowledge within LHD SDH assessment efforts, and provides an example of how to apply PSE and PHCRP to advance an antiracist public health.

## Figures and Tables

**Table 1:** Summary of yHEART^PDX^ training modules for yHEART^PDX^, Vol.I: Youth Participatory Research on Local Social Determinants of Health.

yHEART^PDX^ Training Module	Description
**Public Health 101**	Introduced youth to public health systems and processes in both practice and academic/research contexts. Discussed core aspects of public health laws and regulations, including basic structure of public health powers across legislative, judicial (e.g., case law), and executive branches (e.g., administrative law) of federal, state, and local government. Example discussion topics included aspects of public health law, policy, and practice related to inspections, nuisance abatement, health monitoring and surveillance, quarantine, and environmental regulation. Discussed role of governmental (e.g., CDC, EPA) and non-governmental public health organizations (e.g., NACCHO). Provided a basic overview of core public health subfields of Epidemiology, Health Education/Health Promotion, Environmental Health, Health Systems and Policy, and Biostatistics.
**Epidemiology 101**	Explored historical foundations and evolution of epidemiology, including core elements of modern practice, goals, and tools/approaches. Introduced youth to various areas of epidemiology, including Infections Disease, Chronic Disease, Behavioral, Environmental, and Injury. Discussed the roles, responsibilities, and functions of epidemiology in LHDs. Covered basic definitions for key terms (e.g., “incidence”, “prevalence”, “exposures”, “outcomes”). This session made use of local epidemiology data (e.g., mortality data) to discuss aspects of local health surveillance and monitoring.
**SDH + Social Epi 101**	Developed youth knowledge and understanding of SDH and introduced socioecological models and health equity frameworks. This session made use of local SDH and health data and maps to discuss the impact and role of SDH locally. Discussed basic foundations of social epidemiology (in contrast to traditional epidemiology), including political economy, ecosocial theory, and the notion of embodiment. Highlighted role of structural forms of oppression and exclusion (e.g., racism, class inequality, sexism) in shaping health. Highlighted the “place” and health subfield as an area important for addressing SDH.
**Health Equity 101**	Introduced conceptual foundations and frameworks related to health equity and social justice within public health, and presented basic definitions for critical concepts (e.g., “disparities” vs. “inequities”, health in all policies). Highlighted the role of SDH and population-focused approaches to public health.
**Public Health Research + Research Ethics 101**	Introduced youth to basic elements of public health research, research methods (e.g., quantitative, qualitative, mixed), functions/roles of public health research (e.g., community assessment, accountability, implications for policy), and goals (e.g., addressing health inequities). Additionally, discussed historical foundations and considerations related to research ethics (e.g., Belmont Report, Tuskegee “study”), specifically in relation to human subjects research and research involving vulnerable populations.
**CBPR + YPAR 101**	Introduced core principles of CBPR and YPAR and provided an overview of the potential benefits of participatory research in comparison to traditional research. Highlighted importance of power relations and building community capacity. Additionally, introduced critical concepts of decolonizing research and methods, as well as feminist and Black feminist notions of situated knowledge(s) and centering the margins. Session also covered Freire’s notion of critical consciousness, Gramsci’s notion of “organic intellectual”, and general discussion of popular epidemiology, citizen science, and co-production of knowledge.
**Place, Placemaking, & Health 101**	Developed youth knowledge and understanding of the significance of “place” in shaping health, including notions and mechanisms of “placemaking”. This included introduction to historic and current forces that shape neighborhood built, natural, and social environments, including discussion of core racialized placemaking processes as follows: Indian Removal Act of 1830, Homestead Act of 1862, Oregon Donation Lands Claim Act of 1850, redlining, racially restrictive covenants, the GI Bill, Federal Highway Act of 1956, Gentrification, Serial Forced Displacement, Blockbusting, and exclusionary zoning. This session also discussed general aspects of city planning, land use, and community development. Session was connected to local/regional social determinants of health, making use of local health data and maps to discuss the impact of placemaking processes on SDH locally.

## Data Availability

Not applicable.
